# Liver Scores in the Prognostication of COVID-19 Patients

**DOI:** 10.3390/v17030444

**Published:** 2025-03-19

**Authors:** Thilo Gambichler, Dominic König, Nadine Schuleit, Laura Susok, Wolfgang Schmidt, Nessr Abu Rached

**Affiliations:** 1 Department of Dermatology, Ruhr-University Bochum, 44791 Bochum, Germany; dominic.koenig@rub.de (D.K.); laura.susok@klinikumdo.de (L.S.); nessr.aburached@rub.de (N.A.R.); 2 Department of Dermatology, Christian Hospital Unna, 59423 Unna, Germany; 3 Department of Dermatology, Dortmund Hospital gGmbH, University Witten/Herdecke, 44137 Dortmund, Germany; 4 Department of Internal Medicine, Ruhr-University Bochum, 44791 Bochum, Germany; w.schmidt@klinikum-bochum.de

**Keywords:** SARS-CoV-2, hepatitis, systemic immune-inflammation, hepatic insufficiency, fibrosis, coagulation, comorbidities

## Abstract

The implementation of easily accessible prognostic biomarkers for patients with COVID-19 remains an important area of clinical research. In this large monocentric study at a German tertiary care hospital, we determined the prognostic performance of different liver scores in 605 patients with COVID-19. We evaluated the Fibrosis-4 (FIB-4) index, the Aspartate Aminotransferase-to-Platelet Ratio Index (APRI), the Model for End-Stage Liver Disease (MELD) score, and the De Ritis ratio (DRR; AST/ALT ratio). The 30-day mortality was used as primary COVID-19 outcome measure. The need for intensive care unit (ICU) treatment and overall mortality were secondary endpoints. Univariable analyses showed that most of the investigated liver-related scores (FIB-4, MELD, and DRR), but not APRI for overall mortality, were significantly associated with key outcomes in COVID-19 patients. Concurrently, well-known risk factors—such as advanced age, diabetes, and cardiac or pulmonary comorbidities—were also linked to worse outcomes, except for the female sex having a preventive effect against ICU admission. A history of liver disease was rarely documented among the patients and showed no significant impact on the examined endpoints. Multivariable analyses further revealed that advanced age, DRR, and MELD were independent predictors of both 30-day and overall mortality, while FIB-4 emerged as an independent predictor specifically for overall mortality. Regarding ICU admission, obesity, underlying lung disease, and elevated APRI and MELD scores were identified as independent risk factors, whereas the female sex appeared to be protective. Overall, MELD demonstrated the strongest prognostic value for mortality and ICU admission, with DRR also exhibiting independent predictive power for mortality. These findings suggest that scores originally developed for chronic liver disease assessment—namely FIB-4, APRI, MELD, and DRR—hold promise as prognostic tools in COVID-19. In particular, MELD and DRR emerged as the most powerful biomarkers for predicting severe disease and mortality, highlighting the potential for incorporating these indices into risk stratification models for COVID-19 management. Further prospective multicenter studies are warranted to confirm these observations.

## 1. Introduction

Throughout the COVID-19 pandemic and in the period that followed, healthcare professionals and researchers have worked to identify reliable biomarkers and scoring tools to predict patient outcomes. Various clinical factors, such as age and age-related conditions like lung diseases, cardiovascular disorders, and diabetes mellitus, along with elevated laboratory markers—including C-reactive protein, ferritin, D-dimer, lactate dehydrogenase, lymphocyte counts, and absolute eosinopenia—have been consistently associated with severe COVID-19 cases [1,2,3,4,5]. These markers are linked to complications such as pneumonia, the need for intensive care, or increased mortality risk. In addition to these well-established predictors, liver-related scoring systems originally developed for evaluating chronic liver disease and cirrhosis have gained attention in the context of COVID-19. Examples include the Fibrosis-4 (FIB-4) index, the Aspartate Aminotransferase-to-Platelet Ratio Index (APRI), the Model for End-Stage Liver Disease (MELD) score, and the De Ritis ratio (DRR), which is calculated as the AST/ALT ratio. These scores integrate parameters such as liver enzymes, platelet count, creatinine, and the international normalized ratio (INR) to reflect not only liver function, but also systemic processes such as inflammation, coagulopathy, and multi-organ dysfunction, which play a role in severe cases of COVID-19. While these tools were not initially intended for COVID-19 prognosis, emerging research suggests they might offer valuable insights regarding disease progression. Of these liver scores, the FIB-4 index has been the focus of multiple studies in COVID-19 patients [6,7,8,9,10,11,12,13,14,15,16,17,18,19,20,21,22]. However, there remains a gap in research comparing these liver-related markers collectively within a single, large patient cohort. To address this gap, we conducted a comprehensive monocentric study that simultaneously analyzed FIB-4, APRI, MELD, and DRR—along with potential confounding factors—to evaluate their predictive value in patients infected with SARS-CoV-2.

## 2. Materials and Methods

### 2.1. Patients

This retrospective/prospective monocentric study consecutively recruited COVID-19 patients from the beginning of 2020 to the end of 2022 at a tertiary care hospital (St. Josef) of the Ruhr-University Bochum (Bochum, Germany). Patients with laboratory-confirmed COVID-19 were included in this study (ethics approval: #20-6953-bio). SARS-CoV-2 detection was carried out using a commercial qPCR assay on nasopharyngeal swab specimens (AllplexTM 2019-nCoV, Seegene, Republic of Korea) according to standard protocols. Pregnant women, children and adolescents (age < 16 years), or those whose outcome was unknown were excluded from this study.

### 2.2. Data Extraction and Outcome Measures

All data of SARS-CoV-2-positive patients were extracted from electronic medical files. These data included patient characteristics, relevant comorbidities, laboratory data, and clinical outcomes. Further details are provided in Table 1. COVID-19 30-day mortality was the primary endpoint of this study. As secondary measures of outcome, we included ICU admission and overall COVID-19-associated death. All outcome measures were based on data easily extracted from standard electronic medical files as well as follow-up information provided by patients and physicians.

### 2.3. Liver Scores

All liver scores were determined at baseline in conjunction with COVID-19 diagnosis. MELD score was calculated using bilirubin, creatinine, and the international normalized ratio (INR) with the formula 9.57 × loge (creatinine) + 3.78 × loge (total bilirubin) + 11.2 × loge (INR) + 6.43 [7]. FIB-4 index = [age × aspartate aminotransferase (AST) level (IU/L)]/[platelet count (×10^9^/L)] × [√alanine transaminase (ALT) level (IU/L)]; APRI = AST level (/upper limit of normal range) × 100/platelet count (×10^9^/L); DRR was the AST/ALT ratio.

### 2.4. Statistics

For statistical analysis, MedCalc (Ostend, Belgium) software version 23.1.1 was used. Analysis of data distribution was performed using the D’Agostino–Pearson test. Univariable statistics included the Chi^2^ test for dichotomized data and receiver operating characteristic (ROC) analyses for continuous data [including the associated criterion, area under the curve (AUC), and the Youden index (optimal cut-off points of both the maximum sensitivity and specificity)]. The 30-day mortality timeframe was considered for the analysis using Cox proportional-hazards regression and Kaplan–Meier curves. Multivariable testing was performed using logistic regression, which only included data with significance from univariate testing, specifically a significant AUC of ≥0.60 on ROC analysis or significance with Chi^2^ analysis. ROC curves were also used for goodness of fit of the logistic regression models. As required, the variables included for testing independence did not strongly correlate (*r* < 0.80) with each other. Harrell’s C-index was used as a goodness-of-fit measure for the Cox regression model. For regression analyses, dichotomized/categorical data were used. Odds ratios (ORs) as well as hazard ratios (HRs) including 95% confidence intervals (CIs) were calculated as well; *p* < 0.05 was considered statistically significant.

## 3. Results

### 3.1. Patient Characteristics and Outcome Measures

As shown in Table 1, a total of 605 qPCR-confirmed COVID-19 patients were included in this study (287 females and 318 males). The median age was 60 years (range: 16–97 years). Almost all patients (581, 96%) were at least hospitalized for one day. The laboratory parameters obtained on admission are detailed in Table 2. In this cohort, 128 of 605 (21.2%) patients required treatment in the ICU.

### 3.2. Distribution of Liver Scores at Baseline

As shown in Table 2, all the baseline liver scores investigated were non-normally distributed. The median (range) MELD was 7.29 (5.12–37.53), FIB-4 1.81 (0–31.39), APRI 0.38 (0–20.47), and DRR 1.25 (0–6.68).

### 3.3. Univariable Analysis

As shown in Table 3, we observed several significant variables concerning the primary endpoint, 30-day mortality. Age (AUC 0.82, *p* < 0.0001, criterion: >72, Youden index: 0.53), comorbidities such diabetes (*p* = 0.0434) and cardiovascular diseases (*p* < 0.001), APRI (AUC 0.63, *p* < 0.0050, criterion: >0.60, Youden index: 0.28), DRR (AUC 0.73, *p* < 0.0001, criterion: >1.55, Youden index: 0.43), FIB-4 (AUC 0.76, *p* < 0.0001, criterion: >3.17, Youden index: 0.42), and MELD (AUC 0.78, *p* < 0.0001, criterion: >8.77, Youden index: 0.49) were significantly associated with the 30-day mortality of COVID-19 patients. The overall mortality was significantly associated with age AUC 0.81, *p* < 0.0001, criterion: >73, Youden index: 0.52), diabetes (*p* = 0.034), cardiovascular diseases (*p* < 0.0001), lung diseases *p* = 0.0026, two or more comorbidities *(p* < 0.0001), DRR (AUC 0.74, *p* < 0.000, criterion: >1.30, Youden index: 0.40), FIB-4 (AUC 0.78, *p* < 0.0001, criterion: >3.17, Youden index: 0.45), and MELD (AUC 0.77, *p* < 0.000, criterion: >8.46, Youden index: 0.46). The need for ITU treatment was significantly associated with age (AUC 0.60, *p* = 0.0092, criterion: >52, Youden index: 0.15), diabetes (*p* < 0.0001), obesity (*p* = 0.0001), cardiovascular diseases (*p* < 0.017), lung diseases (*p* < 0.0001), two or more comorbidities (*p* = 0.0007), APRI (AUC 0.64, *p* < 0.0001, criterion: >0.48, Youden index: 0.29), DRR (AUC 0.61, *p* = 0.0001, criterion: >1.44, Youden index: 0.19), FIB-4 (AUC 0.67, *p* < 0.0001, criterion: >2.22, Youden index: 0.27), and MELD (AUC 0.61, *p* = 0.0003, criterion: >9.24, Youden index: 0.20).

### 3.4. Multivariable Analysis

Using the Cox proportional-hazards regression model for multivariable analysis, the following parameters emerged as independent predictors of 30-day mortality: age greater than 72 years [*p* = 0.0004; HR 4.52 (95% CI 1.6 to 10.45)], DRR [*p* = 0.0064; HR 2.40 (95% CI 1.29 to 4.50)], and MELD [*p* = 0.0078; HR 2.69 (95% CI 1.30 to 5.58, Table 4)]. Harrell’s C-index was high, at 0.89 (95% CI 0.82 to 0.89). When overall mortality was used as an outcome measure in the logistic regression model, age greater than 73 years [*p* = 0.0001; OR 4.17 (95% CI 2.01 to 8.62)], DRR [*p* = 0.0064; OR 2.72 (95% CI 1.32 to 5.58)], FIB-4 [*p* = 0.0041; OR 2.53 (95% CI 1.34 to 4.75)], and MELD [*p* = 0.011; OR 2.41 (95% CI 1.23 to 4.74)] remained in the logistic regression model as significant independent predictors. The need for ITU treatment was independently predicted by sex (*p* < 0.0001; OR 0.38 (95% CI 0.23 to 0.60), overweight/obesity [*p* = 0.0001; OR 2.87 (95% CI 1.67 to 4.88)], lung diseases [*p* = 0.0021; OR 2.30 (95% CI 1.35 to 3.90)], APRI [*p* = 0.0096; OR 2.00 (95% CI 1.18 to 3.39)], and MELD [*p* = 0.046; OR 1.67 (95% CI 1.01 to 2.77)]. Subgroup analysis only including patients with ICU treatment (*n* = 128) revealed that a higher MELD [*p* = 0.012; HR 3.39 (95% CI 1.35 to 8.73)] and older age [*p* = 0.0024; 4.88 (95% CI 1.75 to 13.62)] were significant predictors for 30-day mortality. Moreover, DRR [*p* = 0.031; OR 6.38 (95% 1.18 to 34.48)] was the only significant predictor for overall mortality in ICU patients.

The ROC of the logistic regression model for overall mortality was very good, with an AUC of 0.87 (95% CI 0.84 to 0.90). The ROC of the logistic regression model for ITU treatment was good, with an AUC of 0.76 (95% CI 0.72 to 0.79).

## 4. Discussion

While the primary purpose of FIB-4, APRI, MELD, and the DRR is to assess liver disease severity rather than viral or respiratory pathology, multiple retrospective and observational studies suggest a correlation between abnormal liver scores and worse outcomes in COVID-19 [6,7,8,9,10,11,12,13,14,15,16,17,18,19,20,21,22]. Potential reasons include the following: (1) COVID-19 may induce a broad inflammatory response. Liver enzymes may become elevated as part of the “cytokine storm” or as a consequence of endothelial injury and microthromboses; (2) patients with pre-existing liver disease often have a compromised hepatic reserve, making them more vulnerable to COVID-19-related complications; (3) scores that reflect liver injury, coagulopathy, or renal function (like MELD) might capture the broader systemic impact of severe COVID-19. Moreover, the aforementioned liver scores have also shown prognostic power in patients with cancers, including melanoma and Merkel cell carcinoma [23,24].

In COVID-19, elevated FIB-4 values have been associated in some studies with increased severity of disease. The rationale is that FIB-4 incorporates both hepatic injury markers (AST and ALT) and platelet counts—which may reflect systemic inflammation, coagulopathy, or portal hypertension. When patients with COVID-19 demonstrate abnormally high FIB-4 scores, this can indicate worse liver involvement and potentially a higher risk of complications [7,9,10,11,12,13,14]. Similarly to FIB-4, the APRI score was also developed for evaluating fibrosis in viral hepatitis. It uses AST and platelet count to provide a quick assessment of hepatic injury severity. In the context of COVID-19, elevated APRI values appear to correlate with more extensive hepatic dysfunction or inflammation and can be linked to poorer outcomes. While APRI alone may not be a definitive marker for predicting mortality or respiratory failure, it does offer a simple, readily available method for flagging patients who may require closer monitoring or more aggressive therapeutic strategies [9,14,25].

The MELD score is a well-known tool used in patients with cirrhosis to prioritize liver transplantation candidates by estimating short-term mortality risk [15,16,17,18]. It incorporates bilirubin, creatinine, and the INR (international normalized ratio). In COVID-19 patients—particularly those with pre-existing liver disease—an elevated MELD score can highlight both baseline and evolving liver dysfunction, which in turn may worsen overall prognosis. Because MELD also reflects renal function (via creatinine) and coagulopathy (via INR), it can capture broader systemic derangements that occur in severe COVID-19 [15,16,17,18]. DRR, or simply the AST/ALT ratio, has long been used to differentiate etiologies of hepatic injury, such as alcoholic versus non-alcoholic liver damage [19,20,21,22]. A ratio > 2 often suggests alcoholic liver disease, for instance. In COVID-19, a higher DRR could indicate more profound hepatocellular stress or reflect multisystemic injury affecting the liver. Although less comprehensive than FIB-4, APRI, or MELD, an abnormal DRR in a patient with COVID-19 could serve as an early warning sign for hepatic involvement or severe disease progression [19,20,21,22].

This study represents the first instance where the prognostic performance of four different liver-related scores—FIB-4, APRI, MELD, and DRR—were evaluated within the same population, allowing for direct comparison. The univariable analysis indicated that all examined scores, except APRI, were significantly associated with clinical outcomes in COVID-19 patients, including 30-day mortality, overall mortality, and the need for intensive care unit (ICU) admission. Additionally, established risk factors for COVID-19, such as advanced age, diabetes, and cardiovascular or pulmonary conditions, showed significant correlations with both primary and secondary outcomes. In the present study, the history of liver diseases was very low and not associated with the outcome measures investigated. Multivariable analysis revealed that advanced age, DRR, and MELD independently predicted both 30-day and overall mortality. FIB-4 was identified as an independent predictor specifically for overall mortality. Other clinical factors, including sex, obesity, and lung diseases, did not retain significance in the regression models. ICU admission was independently linked to obesity, pre-existing lung diseases, and elevated APRI and MELD scores, while the female sex appeared as a strong protective factor against ICU admission. Overall, MELD emerged as the most reliable predictor for both mortality and ICU admission. DRR also demonstrated independent predictive value for mortality in patients with COVID-19. In comparison, FIB-4 and APRI exhibited weaker performance in predicting important outcomes of COVID-19 patients.

The limitations and strengths of the present study include the following: (1) our mixed retrospective/prospective analysis was limited by the data collected in a real-world clinical environment, potentially leading to information bias; (2) clinical judgments by treating clinicians directed data collection, possibly resulting in missing data and incomplete analyses; in particular, verification of underlying liver conditions was not performed; (3) given the overwhelming workload and pressure stressing the healthcare system during the first COVID-19 infection waves, we analyzed all biomarkers only at the time of hospital admission and not longitudinally; (4) co-infection with other pathogens and comorbidities may also be confounding factors for this study with regard to COVID-19 mortality. However, the strengths of the present study include a reasonable sample size and the fact that several liver scores were analyzed and correlated with three different outcome measures.

## 5. Conclusions

In summary, liver scores such as FIB-4, APRI, MELD, and the DRR—though originally developed for assessing chronic liver disease—have shown potential prognostic implications in COVID-19. At least in univariable analysis, elevated values in almost all of these indices may signal an increased risk for severe disease and poor outcomes. In this comparative investigation using multivariable analyses, however, we found that MELD and DRR are the most powerful biomarkers in predicting meaningful outcomes such as severe disease and mortality. Further prospective multicenter studies will help to clarify their precise role and refine their utility in the management of COVID-19 patients.

## Figures and Tables

**Table 1:** Descriptive clinical baseline data and outcomes of COVID-19 patients (*n* = 605) treated in a German tertiary care hospital (part (**a**)). Comorbidities * of COVID-19 patients are shown in part (**b**).

(a)		(b)	
Parameter	Data	Parameter	Data
**Sex**		Overweight/obesity ***	
Female/male	287/318 (47.4%/52.6%)	no/yes	324/88 (78.6%/21.4%)
**Median (range) age**		**Diabetes mellitus**	
(years)	60 (16–97)	no/yes	474/131 (78.3%/21.7%)
**Intensive care unit**		**Lung diseases**	
no/yes	477/128 (78.8%/21.2%)	no/yes	480/125 (79.3%/20.7%)
30-day mortality **		**Cardiovascular diseases**	
no/yes	553/52 (91.4%/8.6%)	no/yes	307/298 (50.7%/49.3%)
30-day mortality **		**Liver diseases**	
median (range)	12.5 (1–30)	no/yes	587/18 (97%/3%)
Overall mortality **		**At least two comorbidities**	
no/yes	539/66 (89.1%/10.9%)	no/yes	340/265 (56.2%/43.8%)

* previously known/documented comorbidities; ** associated with COVID-19; *** overweight/obesity was defined as a body mass index > 25 kg/m^2^.

**Table 2:** Baseline laboratory data of liver scores of COVID-19 patients treated in a German tertiary care hospital.

Parameter	MELD	FIB-4	APRI	DRR
Median (range)	7.29 (5.12–37.53)	1.81 (0–31.39)	0.38 (0–20.47)	1.25 (0–6.68)
“Normal” range	6	1.3	0.3	0.7–1.2

**Table 3:** Univariable analysis including receiver operating curves (ROCs) and Chi^2^ tests in order to determine significant prognostic biomarkers for the outcomes of patients with COVID-19. We exclusively included parameters revealing a significant AUC ≥ 0.60 on ROC analysis or a statistically significant result on the Chi^2^ test.

Parameter	30-Day Mortality *	Overall Mortality *	Intensive Care Unit
**Age**	AUC 0.82, *p* < 0.0001 Criterion: >72, Youden index: 0.53	AUC 0.81, *p* < 0.0001 Criterion: >73, Youden index: 0.52	AUC 0.60, *p* = 0.0092 Criterion: >52, Youden index: 0.15
**Sex**	-	-	*p* = 0.0009
**Diabetes**	*p* = 0.0434	*p* = 0.034	*p* < 0.0001
**Overweight/obesity**	-	-	*p* = 0.0001
**Cardiovascular diseases**	*p* < 0.001	*p* < 0.0001	*p* < 0.017
**Lung diseases**	-	*p* = 0.0026	*p* < 0.0001
**Two or more comorbidities**	-	*p* < 0.0001	*p* = 0.0007
**APRI**	AUC 0.63, *p* < 0.0050 Criterion: >0.60, Youden index: 0.28	-	AUC 0.64, *p* < 0.0001 Criterion: >0.48, Youden index: 0.29
**DRR**	AUC 0.73, *p* < 0.0001 Criterion: >1.55, Youden index: 0.43	AUC 0.74, *p* < 0.0001 Criterion: >1.30, Youden index: 0.40	AUC 0.61, *p* = 0.0001 Criterion: >1.44, Youden index: 0.19
**FIB-4**	AUC 0.76, *p* < 0.0001 Criterion: >3.17, Youden index: 0.42	AUC 0.78, *p* < 0.0001 Criterion: >3.17, Youden index: 0.45	AUC 0.67, *p* < 0.0001 Criterion: >2.22, Youden index: 0.27
**MELD**	AUC 0.78, *p* < 0.0001 Criterion: >8.77, Youden index: 0.49	AUC 0.77, *p* < 0.0001 Criterion: >8.46, Youden index: 0.46	AUC 0.61, *p* = 0.0003 Criterion: >9.24, Youden index: 0.20

* associated with COVID-19.

**Table 4:** Multivariable analyses (Cox proportional-hazards regression and logistic regression models) included dependent variables, such as COVID-19-associated 30-day mortality, overall mortality, and the need for intensive care unit treatment of 605 COVID-19 patients. Independent variables were included in the model if there was a significant AUC ≥ 0.60 on ROC analysis or statistically significant results on Chi^2^ testing (Table 3).

Parameter	30-Day Mortality *	Overall Mortality *	Intensive Care Unit
**Age**	*p* = 0.0004; HR 4.52 (95% CI 1.6 to 10.45)	*p* = 0.0001; OR 4.17 (95% CI 2.01 to 8.62)	-
**Sex**	-	-	*p* < 0.0001; OR 0.38 (95% CI 0.23 to 0.60)
**Overweight/obesity**	-	-	*p* = 0.0001; OR 2.87 (95% CI 1.67 to 4.88)
**Lung diseases**	-	-	*p* = 0.0021; OR 2.30 (95% CI 1.35 to 3.90)
**APRI**	-	-	*p* = 0.0096; OR 2.00 (95% CI 1.18 to 3.39)
**DRR**	*p* = 0.0064; HR 2.40 (95% CI 1.29 to 4.50)	*p* = 0.0064; OR 2.72 (95% CI 1.32 to 5.58)	-
**FIB-4**	-	*p* = 0.0041; OR 2.53 (95% CI 1.34 to 4.75)	-
**MELD**	*p* = 0.0078; HR 2.69 (95% CI 1.30 to 5.58)	*p* = 0.011; OR 2.41 (95% CI 1.23 to 4.74)	*p* = 0.046; OR 1.67 (95% CI 1.01 to 2.77)

* related to COVID-19.

## Data Availability

The data that support the findings of this study are available from the corresponding author upon reasonable request.

## References

[1] Tian W., Jiang W., Yao J., Nicholson C.J., Li R.H., Sigurslid H.H., Wooster L., Rotter J.I., Guo X., Malhotra R. (2020). Predictors of mortality in hospitalized COVID-19 patients: A systematic review and meta-analysis. J. Med. Virol..

[2] Li J., Huang D.Q., Zou B., Yang H., Hui W.Z., Rui F., Yee N.T.S., Liu C., Nerurkar S.N., Kai J.C.Y. (2021). Epidemiology of COVID-19: A systematic review and meta-analysis of clinical characteristics, risk factors, and outcomes. J. Med. Virol..

[3] Al-Shajlawi M., Alsayed A.R., Abazid H., Awajan D., Al-Imam A., Basheti I. (2022). Using laboratory parameters as predictors for the severity and mortality of COVID-19 in hospitalized patients. Pharm. Pract..

[4] Gambichler T., Schuleit N., Susok L., Becker J.C., Scheel C.H., Torres-Reyes C., Overheu O., Reinacher-Schick A., Schmidt W. (2023). Prognostic Performance of Inflammatory Biomarkers Based on Complete Blood Counts in COVID-19 Patients. Viruses.

[5] Notarte K.I., de Oliveira M.H.S., Peligro P.J., Velasco J.V., Macaranas I., Ver A.T., Pangilinan F.C., Pastrana A., Goldrich N., Kavteladze D. (2022). Age, Sex and Previous Comorbidities as Risk Factors Not Associated with SARS-CoV-2 Infection for Long COVID-19: A Systematic Review and Meta-Analysis. J. Clin. Med..

[6] Kamiya Y., Shinoda M., Ishii N., Yamamoto S., Sekine T., Morikawa M., Ota S., Toyama-Kousaka M., Takahashi H., Takei H. (2024). Comparison of liver fibrosis scores and fatty liver on computed tomography as risk factors for severity of COVID-19. JGH Open.

[7] Lucena Valera A., Aller de la Fuente R., Sánchez Torrijos Y., Romero Gómez M., Ampuero Herrojo J. (2024). FIB-4 score as a predictor of COVID-19-related severity in hospitalized patients. Rev. Esp. Enferm. Dig..

[8] Mendoza-Hernandez M.A., Hernandez-Fuentes G.A., Sanchez-Ramirez C.A., Rojas-Larios F., Guzman-Esquivel J., Rodriguez-Sanchez I.P., Martinez-Fierro M.L., Cardenas-Rojas M.I., De-Leon-Zaragoza L., Trujillo-Hernandez B. (2024). Time-dependent ROC curve analysis to determine the predictive capacity of seven clinical scales for mortality in patients with COVID-19: Study of a hospital cohort with very high mortality. Biomed Rep..

[9] Çopur B., Sürme S., Tunçer G., Bayramlar O.F. (2023). The Role of APRI, FIB-4, and SAD-60 Scores as Predictors of Mortality in COVID-19 Patients. Infect. Dis. Clin. Microbiol..

[10] Parajuli P., Sabo R., Alsaadawi R., Robinson A., French E., Sterling R.K. (2023). Fibrosis-4 (FIB-4) index as a predictor for mechanical ventilation and 30-day mortality across COVID-19 variants. J. Clin. Transl. Sci..

[11] Miele L., Dajko M., Savino M.C., Capocchiano N.D., Calvez V., Liguori A., Masciocchi C., Vetrone L., Mignini I., Schepis T. (2023). Fib-4 score is able to predict intra-hospital mortality in 4 different SARS-CoV-2 waves. Intern. Emerg. Med..

[12] Ao G., Li A., Li J., Du X., Wang Y., Tran C., Qi X. (2022). The Association Between the Fibrosis-4 Index and COVID-19: A Systematic Review and Meta-Analysis. Ann. Clin. Lab. Sci..

[13] Lombardi R., Mura V., Cespiati A., Iuculano F., Sigon G., Pallini G., Proietti M., Motta I., Montinaro B., Fiorelli E. (2022). Usefulness of fibrosis-4 (FIB-4) score and metabolic alterations in the prediction of SARS-CoV-2 severity. Intern. Emerg. Med..

[14] Grigoras M.L., Citu I.M., Citu C., Chiriac V.D., Gorun F., Levai M.C., Manolescu D., Rosca O., Bratosin F., Gurumurthy S. (2022). Evaluation of FIB-4, NFS, APRI and Liver Function Tests as Predictors for SARS-CoV-2 Infection in the Elderly Population: A Matched Case-Control Analysis. J. Clin. Med..

[15] Reyes-Ruiz J.M., Avelino-Santiago A.C., Martínez-Mier G., López-López C.V., De Jesús-González L.A., León-Juárez M., Osuna-Ramos J.F., Farfan-Morales C.N., Palacios-Rápalo S.N., Bernal-Dolores V. (2024). The Model for End-Stage Liver Disease (MELD) Score Predicting Mortality Due to SARS-CoV-2 in Mexican Patients. J. Clin. Med..

[16] Kaya Y., Gülcü O., Aksakal E., Kalkan K., Aydın S.Ş., Kaya A., Bostan S. (2023). A significant predictor of in-hospital and long-term mortality and progression in COVID-19 patients: The end-stage liver disease (MELD) score model. J. Med. Virol..

[17] Stawinski P.M., Dziadkowiec K.N., Al-Abbasi B., Suarez L., Simms L., Dewaswala N., Torres P., Al Rubaye A., Pino J., Marcus A. (2021). Model of End-Stage Liver Disease (MELD) Score as a Predictor of In-Hospital Mortality in Patients with COVID-19: A Novel Approach to a Classic Scoring System. Cureus.

[18] Wagner J., Garcia-Rodriguez V., Yu A., Dutra B., Bhatt A., Larson S., Farooq A. (2020). The Model for End-Stage Liver Disease-Sodium Score at Admission Is Prognostic of Covid-19 Disease Severity. SN Compr. Clin. Med..

[19] Raj K., Kumari B., Kumar S., Bansal A., Mohanraj P.S., Dey S., Thakur A. (2024). The Prognostic Value of De Ritis Ratio on the Survival Rate of ICU-Admitted COVID-19 Patients During the Second Wave: A Retrospective Study in a Tertiary Care Hospital in India. Cureus.

[20] Mangoni A.A., Zinellu A. (2023). An Updated Systematic Review and Meta-Analysis of the Association between the De Ritis Ratio and Disease Severity and Mortality in Patients with COVID-19. Life.

[21] Drácz B., Czompa D., Müllner K., Hagymási K., Miheller P., Székely H., Papp V., Horváth M., Hritz I., Szijártó A. (2022). The Elevated De Ritis Ratio on Admission Is Independently Associated with Mortality in COVID-19 Patients. Viruses.

[22] Zinellu A., Arru F., De Vito A., Sassu A., Valdes G., Scano V., Zinellu E., Perra R., Madeddu G., Carru C. (2021). The De Ritis ratio as prognostic biomarker of in-hospital mortality in COVID-19 patients. Eur. J. Clin. Investig..

[23] Gambichler T., Becker J.C., Susok L., Käpynen R., Abu Rached N. (2023). Model for End-Stage Liver Disease Correlates with Disease Relapse and Death of Patients with Merkel Cell Carcinoma. Cancers.

[24] Abu Rached N., Reis M.M.D.S., Stockfleth E., Käpynen R., Gambichler T. (2024). Analysis of Calculated Liver Scores for Long-Term Outcome in 423 Cutaneous Melanoma Patients. Cancers.

[25] Peschel G., Grimm J., Buechler C., Gunckel M., Pollinger K., Aschenbrenner E., Kammerer S., Jung E.-M., Haimerl M., Werner J. (2021). Liver stiffness assessed by shear-wave elastography declines in parallel with immunoregulatory proteins in patients with chronic HCV infection during DAA therapy. Clin. Hemorheol. Microcirc..

